# Successful biodegradable stent insertion in an infant with severe bronchomalacia and cystic fibrosis

**DOI:** 10.1016/j.jcf.2020.10.004

**Published:** 2021-03

**Authors:** Caroline A. Harris, Malcolm Brodlie, Christopher O'Brien, Matthew F. Thomas

**Affiliations:** a Paediatric Respiratory Medicine, Great North Children's Hospital, Newcastle upon Tyne Hospitals NHS Foundation Trust, Newcastle upon Tyne, UK; b Translational and Clinical Research Institute, Faculty of Medical Sciences, Newcastle University, Newcastle upon Tyne, UK

**Keywords:** Cystic fibrosis, Airway, Tracheomalacia, Bronchomalacia, Biodegradable stent

## Abstract

We report the first case of biodegradable airway stent insertion for a patient with bronchomalacia and cystic fibrosis (CF). This female infant with antenatally diagnosed cystic fibrosis developed respiratory distress by three weeks of age. On lower airway examination she was found to have severe left main stem bronchomalacia causing left upper lobe hyperinflation and subsequent right upper lobe collapse. By eight weeks of age she developed life-threatening respiratory failure requiring high pressure invasive ventilation. A biodegradable bronchial stent was inserted to the left main bronchus at thirteen weeks of age with successful extubation from invasive respiratory support four days later. A second biodegradable stent was inserted nine weeks later, due to persistent bronchomalacia following stent degradation and redevelopment of oxygen requirement. She was discharged home off all respiratory support eight weeks later and has remained well, requiring no further surgical intervention for bronchomalacia to date, now age three years.

## Highlights

• A biodegradable bronchial stent was safely used in an infant with CF.
• A biodegradable stent can support airway during growth to overcome bronchomalacia.
• No superadded infection with a biodegradable bronchial stent in an infant with CF.

## 1 Introduction

Cystic fibrosis (CF) is the most common fatal genetic disease in the Caucasian population [1]. Lung disease remains the leading cause of morbidity and mortality in this multisystem disease, with airway infection, inflammation and mucus accumulation as characteristic features [2]. Airway malacia is defined as greater than 50% reduction in the cross-sectional luminal area during quiet expiration [3]. This can occur at any level of the airway as a primary condition or be associated with an underlying condition [3]. The incidence of airway malacia in the general paediatric population has been reported as ranging from 1 in 1400 to 1 in 2,100 [4]. The prevalence of tracheomalacia in children with CF diagnosed bronchoscopically has been reported as 13.9-15.5% [1,4]. In adults with CF the prevalence identified by CT scan has been found to be as high as 69% [5]. This increase in adulthood is thought to be acquired secondary to airway infection and inflammation [1]. However, increasing work in animal models suggests the cystic fibrosis transmembrane conductance regulator (CFTR) may have a role in airway development leading to early airway changes present from birth [2].

We report a case of an infant with CF and life-threatening bronchomalacia who was successfully treated with a biodegradable bronchial stent in order to wean from ventilatory support.

## 2 Case

Our patient was antenatally diagnosed with CF having been found to be homozygous for c.1521_1523delCTT p.(Phe508del) and c.1006_1007insG p.(Ile336fs) following amniocentesis. Anomaly scans also raised concerns of echogenic bowel and polyhydramnios. She was born in good condition at 35 weeks gestation by emergency caesarian section due to concerns on cardiotocograph. Birth weight was 2.46kg (50^th^-75^th^ percentile). A contrast study on day one of life confirmed intestinal obstruction leading to laparotomy. Meconium ileus was confirmed and a large section (approximately 100cm) of necrotic small bowel was excised. A jejunostomy was formed and she was subsequently extubated to air on day five of life. She remained an inpatient whilst establishing enteral feeds.

**Fig. 1:**
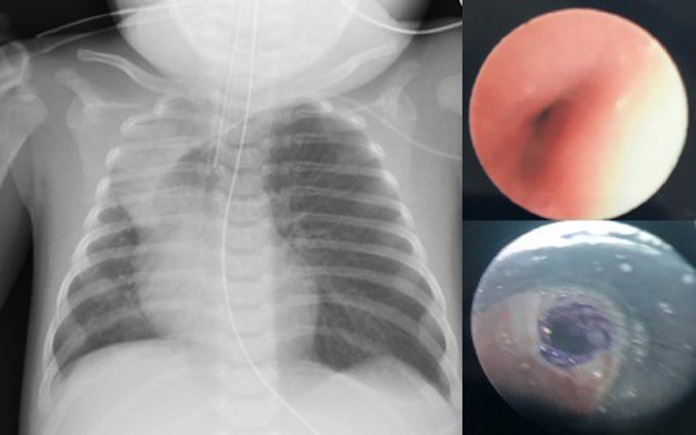
(Left) Chest X-ray taken aged 8 weeks after admission to intensive care. Marked hyperinflation of left upper lobe with collapse of right upper lobe and significant mediastinal shift to the right. (Top right) Image taken from first flexible bronchoscopy showing severe left main stem bronchomalacia. (Bottom right) Image taken immediately after first biodegradable stent insertion to left main bronchus age 13 weeks.

At three weeks of age she developed increased work of breathing and an oxygen requirement. Chest x-ray revealed hyperinflation of the left upper lobe causing mediastinal shift and collapse of the right upper lobe (Fig. 1). Flexible bronchoscopy showed severe slit-like left main stem bronchomalacia (Fig. 1). Echocardiogram confirmed a structurally normal heart. A chest CT showed atelectasis in the right upper lobe on CT with trachea and main bronchi reported to be of normal calibre.

**Fig. 2:**
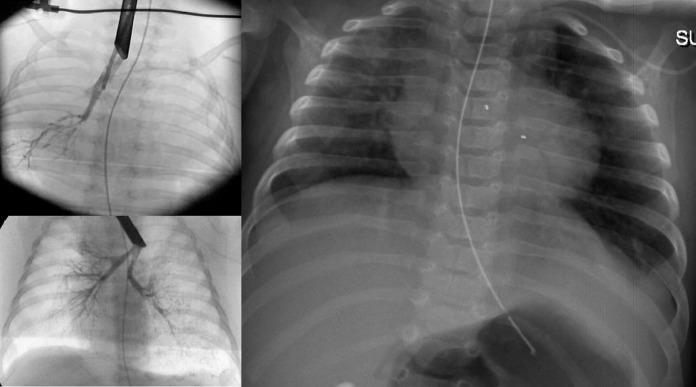
**(**Top left) Bronchogram prior to first stent insertion. (Bottom left) Bronchogram one day following initial stent insertion. (Right) Chest X-ray taken 10 days after stent insertion. Bronchial stent markers visible and nasogastric tube in situ.

**Fig. 3:**
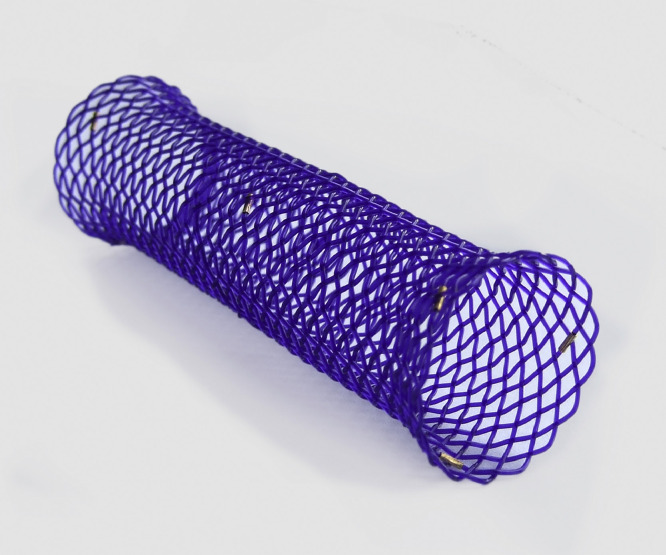
SX-ELLA BD biodegradable stent. (Photograph included with manufacturer's permission).

Respiratory support was increased to high flow nasal cannula (HFNC) oxygen however she developed hypercapnia and hypoxia requiring invasive ventilation by eight weeks of age. Ventilation was difficult to wean with high oxygen requirements and so the decision was made by the multidisciplinary trachea-bronchial airway group and CF team to insert a biodegradable bronchial stent which was expected to maintain integrity for four to six weeks (Fig. 2). This was done at thirteen weeks of age using an Ella-CS custom made stent (5 × 10mm) (Fig. 3). A proactive approach was taken to optimise airway clearance and prevent infection. Mucolytics, dornase alpha and acetylcysteine nebulisers were started as well as empirical cover for *Pseudomonas aeruginosa* with intravenous ceftazidime and tobramycin. She was subsequently extubated to HFNC four days after stent insertion and this was continued at 2 litres/minute to provide airway humidification although she had no oxygen requirement. In total she was invasively ventilated for 31 days. Following stent insertion the only pathogens isolated from respiratory secretions were *Staphylococcus aureus* and Rhino/enterovirus.

Five weeks following stent insertion she developed further respiratory distress and redeveloped an oxygen requirement. Repeat bronchoscopy showed that the stent had migrated and was causing partial blockage of the left upper lobe with proximal bronchomalacia. She remained stable on HFNC until a second biodegradable stent (5 × 20mm) was inserted nine weeks following the initial stent. She continued to make good progress following this and was weaned off all respiratory support 20 days after second stent insertion, and discharged home eight weeks later.

Over the next two years she had intermittent wheeze exacerbated by airway infection, but this has steadily reduced over time. Most recent bronchoscopy was performed age 2 years which showed persisting severe distal left main stem bronchomalacia, however she has not required any admissions for respiratory support since infancy. *Pseudomonas aeruginosa* was isolated age 2.5 years which was successfully eradicated after three months of treatment. Otherwise she has not had an unusual microbiological course to date. Most recent chest x-ray age 3 years appears normal except for some peri-bronchial thickening.

## 3 Discussion

We report the first case, to our knowledge, of biodegradable stent insertion to treat bronchomalacia in a mechanically ventilated infant with CF. A single case is reported of treatment of bronchomalacia by insertion of a removeable silicone stent in a patient with cystic fibrosis. This patient however was fifteen years old and unventilated at the time of stent insertion [6]. We felt that long term intubation and ventilation in the presence of collapse/hyperinflation would result in bacterial overgrowth and progressive lung damage. Tracheostomy insertion was also considered but discounted as intubation and ventilation had not helped the collapse /hyperinflation and loss of upper airway defences would have been an additional burden. Use of a metal stent was ruled out due to the risk of long-term bacterial overgrowth with foreign material in a CF airway and probability of long-term stricture or bronchial erosion. The biodegradeable bronchial stent was able to support this infant until airway growth was adequate enough to overcome severe bronchomalacia and there were no secondary complications of superinfection using this method.

Increasing work in animal models has suggested that the lack of functional CFTR may affect airway development from birth [2]. Newborn piglets with CF showed significantly higher airway resistance, smaller proximal airway lumens and evidence of air trapping compared to controls on day one of life supporting the theory that airway changes in CF may be congenital [2]. Tracheomalacia has been reported as being more prevalent in people with CF, [1,4] and most commonly in children with severe manifestations of CF such as meconium ileus [1]. Important associations of both conditions have been lower FEV_1_ measurements and earlier acquisition of *P. aeruginosa* [1].

## 4 Conclusions

We have shown that biodegradable bronchial stenting can be a successful treatment for life-threatening bronchomalacia without secondary complications of superinfection in a patient with CF. This experience will be of future relevance as tracheobronchomalacia is more common in children with CF than the general paediatric population [1,4].

## Funding

This research did not receive any specific grant from funding agencies in the public, commercial, or not-for-profit sectors. MB was supported by a Medical Research Council Clinician Scientist Fellowship (MR MR/M008797/1).

## Declaration of Competing Interest

None relating to this work. M.B. unrelated to this work, received investigator-led research grants from Pfizer and Roche Diagnostics; honoraria for speaking at educational meetings paid to Newcastle University from Novartis, TEVA and Roche Diagnostics; and travel and accommodation for educational meetings from Boehringer Ingelheim and Vertex Pharmaceuticals. M.F.T. unrelated to this work, received an investigator-led research grant from Pfizer. All remaining authors: No reported conflicts of interest.
